# Crosstalk between fatty acid metabolism and tumour-associated macrophages in cancer progression

**DOI:** 10.37796/2211-8039.1381

**Published:** 2022-12-01

**Authors:** Noorzaileen Eileena Zaidi, Nur Aima Hafiza Shazali, Thean Chor Leow, Mohd Azuraidi Osman, Kamariah Ibrahim, Nik Mohd Afizan Nik Abd Rahman

**Affiliations:** a Department of Cell and Molecular Biology, Faculty of Biotechnology and Biomolecular Sciences, Universiti Putra Malaysia, 43400, Serdang, Selangor, Malaysia; b Institute of Tropical Forestry and Forest Products, Universiti Putra Malaysia, 43400, Serdang, Selangor, Malaysia; c Department of Biomedical Science, Faculty of Medicine, University of Malaya, 50603 Kuala Lumpur, Malaysia

**Keywords:** Fatty acid metabolism, Palmitic acid, Oleic acid, Invasion, Metastasis, Tumour-associated macrophages

## Abstract

Over the last few decades, cancer has been regarded as an independent and self sustaining progression. The earliest hallmarks of cancer comprise of sustaining proliferative signalling, avoiding growth suppressors, resisting cell death, enabling replicative immortality, inducing angiogenesis, and activating invasion and metastasis. Nonetheless, two emerging hallmarks are being described: aberrant metabolic pathways and evasion of immune destruction. Changes in tumour cell metabolism are not restricted to tumour cells alone; the products of the altered metabolism have a direct impact on the activity of immune cells inside the tumour microenvironment, particularly tumour-associated macrophages (TAMs). The complicated process of cancer growth is orchestrated by metabolic changes dictating the tight mutual connection between these cells. Here, we discuss approaches to exploit the interaction of cancer cells’ abnormal metabolic activity and TAMs. We also describe ways to exploit it by reprogramming fatty acid metabolism via TAMs.

## 1. Introduction

The tumour microenvironment (TME) is a complex environment in which cancer cells develop and are driven by an intimate crosstalk of intra- and extracellular compartments, which includes inflammatory cells, fibroblasts, malignant cells, and endothelial cells. Furthermore, the scaffold structure surrounding the cells and the soluble substances secreted by the cells are all mixed in with cancer cells. Extracellular metabolites are also involved in these intricate interactions, which are linked to the development of hypoxia, inadequate nutritional availability, accumulation of metabolic products, and unfavourable pH. All of these parameters have a significant impact on the definition of cancer cell metabolism pathways [1,2]. The cancer- associated metabolites not only provide energy to cancer cells, but also act as communication signals that are tailored to their high energy demands, uncontrolled proliferation, and adaptation to shifting cellular roles and functions [3,4]. In these conditions, the level of tumour metabolic environment may be exploited or hampered by a plethora of mechanisms intrinsically associated to cancer growth, such as colonisation of new metastatic niches and immunosurveillance evasion.

Aside from these elements, inflammatory cells in the microenvironment surrounding the tumour also contribute to cancer growth and progression. In 1863, Rudolph Virchow proposed that inflammation contributes to the development of cancer by implying the presence of infiltrating immune cells at the site of the tumour lesion in the inflamed tissue area [5]. Approximately 50% of the macrophage population has been detected in solid tumours and has been shown to perform an important pro-tumoral role [6,7]. Macrophages are heterogeneous and have a wide range of plasticity and functionality, allowing them to differentiate between separate phenotypes known as M1 (anti-tumoral phenotype) and M2 (pro-tumoral phenotype) depending on microenvironmental stimuli [8,9]. These macrophages are predominantly found infiltrated and differentiated within the TME and are known as “tumour-associated macrophages” or TAMs at all stages of tumour growth [10]. Cancer cells’ bidirectional metabolic interactions with TAMs are hypothesised to contribute to carcinogenesis via altered metabolism metabolites.

In order to meet the increased demand for biosynthesis during high levels of proliferation, cancer cells within the tumour compartment must reprogramme their fatty acid metabolism to conform to metabolic symbiosis. In general, fatty acids (FAs) are macromolecules that participate in a variety of cellular activities, such as providing substrates for energy synthesis [11], developing cell membrane structure [12], and signalling pathway modification [13]. Other studies have demonstrated that FAs have a substantial effect on the development of immune cells, particularly macrophages, as well as their activation and functions, with a considerable effect on the balance of anti- and proinflammatory signals in both homeostatic state and immunological response [14,15].

In this review, we discuss recent findings regarding the roles of FAs, specifically palmitic acid (PA) and oleic acid (OA), as key players in metabolic circuits that allow cancer progression in conjunction with the ability of TAMs to structure the development of tumour microenvironment and address the metabolic stimuli involved in bidirectional communications between cancer cells and TAMs. We also present a platform for developing novel concepts for inhibiting cancer progression by targeting and manipulating FA metabolism in cancer cells.

## 2. Fatty acid uptake/*De novo* fatty acid synthesis in cancer cells and macrophages

Intrinsic and extrinsic signals from cells regulate metabolic pathways to modify the metabolic machinery that regulates the systhesis of key metabolites to fulfil in cellular growth demands. FAs, for instance, are required by highly proliferating cancer cells to maintain cell growth [16], disseminate [17], regulate membrane assembly [18], trigger proliferative signalling [19], and act as bioenergetic needs [20]. In the context of immunity, changes in metabolic pathways influence immune effector activities, most notably the macrophage polarisation and the inflammatory response [21,22].

Nonetheless, Warburg published a crucial work in the 1920s explaining how neoplastic cells prefer glycolytic ATP production, followed by lactate generation as the ultimate product, over mitochondrial oxidation, regardless of the availability of sufficient oxygen [23,24]. The Warburg effect is the name given to this phenomena. In addition, *de novo* fatty acid synthesis is a significant cancer hallmark. FA synthesis is a dynamic interaction of metabolic pathways that is critical for the development of cellular membranes as well as the production of crucial synthetic precursors required for cancer cell growth [25]. The availability of metabolites from other cell intrinsic metabolic pathways, such as glycolytic and tricarboxylic acid (TCA) cycle metabolism, has a significant impact on the end products of FA synthesis.

**Fig. 1:**
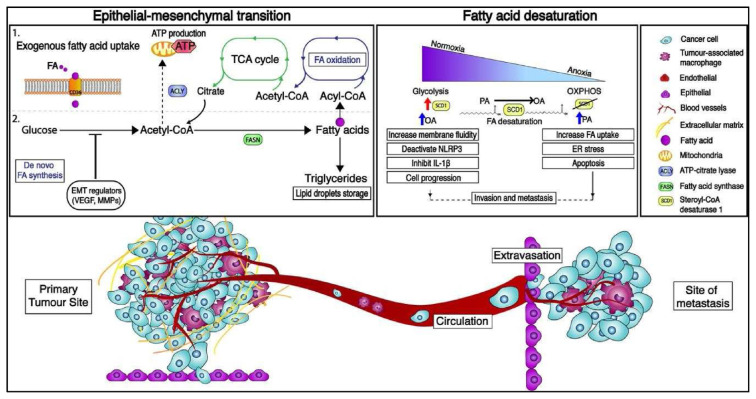
The metabolic characteristics of the epithelial-to-mesenchymal transition (EMT) as well as an overview of the metastatic cascade. Several components of the molecular pathways that drive EMT have a major impact on cell metabolism and vice versa, leading in metabolic rewiring within the glycolysis, tricarboxylic acid (TCA) cycle, and fatty acid production pathways. Exogenous absorption and de novo lipogenesis are two methods by which cancer cells obtain fatty acids (FAs). CD36, a specialised transporter, facilitates exogenous FA uptake from the environment. FAs can then be stored as lipid droplets and integrated into triglycerides for energy storage, as well as used to produce acetyl-coA via FA-oxidation. Cancer cells also use glucose as a carbon source for de novo lipogenesis in order to synthesise citrate. The enzymatic activity of ATP-citrate lyase (ACLY), fatty acid synthase (FASN), and acyl-coA carboxylase produce FAs from citrate (ACC). Furthermore, depending on the oxygen tension, EMT-committed cancer cells may rely on an aerobic glycolytic metabolism or shift toward oxidative phosphorylation (OXPHOS). The suppression of stearoyl-CoA desaturase 1 (SCD1) in cancer cells produces endoplasmic reticulum (ER) stress and apoptosis, demonstrating that decreasing oxygen tension causes changes in metabolism via fatty acid desaturation. VEGF stands for vascular endothelial growth factor, MMP stands for matrix metalloproteinases, and NLRP3 stands for NLR family pyrin domain containing 3.

The FA synthesis begins with the conversion of citrate to acetyl-Coenzyme A (acetyl-CoA), which is then expanded into malonyl-CoA [26]. The *de novo* FA synthesis pathway was catalysed by many enzymatic pathways including two key enzymes, acetyl-CoA carboxylase (ACC) and fatty acid synthase (FASN) (Fig. 1). Thereafter, FASN elongates the nascent FA chain in a nicotinamide adenine dinucleotide phosphate (NADPH)-dependent manner until saturated fatty acid (SFA) palmitate (PA) is synthesized. FAs generated by FASN are integrated into lipid droplets as triglycerides (TAGs) for energy storage and subsequently catabolized by FA oxidation (FAO) [27].

Cancer cells secrete FAs in a unique way, activating the *de novo* or endogenous FA synthesis pathway with resources from the local microenvironment such as glutamine and glucose [28]. Exogenous food lipids are preferentially utilised to synthesise new structural lipids in normal human tissue, but *de novo* FA-synthesis is frequently inhibited, and FASN expression remains low. On the contrary, *de novo* FA synthesis is optimised and frequently increased in cancer cells. To maintain their rapid proliferative rate and provide vital energy for metabolic processes, cellular FA supply is fully dependent on *de novo* synthesis.

Alternatively, the carnitine shuttle system transports exogenous fatty acids into the mitochondrion via specialised transporters [29]. FAO is a multistep catabolic process that converts exogenous long-chain FAs into acetyl-CoA, which is then oxidised via the TCA cycle and the electron transport chain (ETC) chain to generate ATP (Fig. 1). CD36, a scavenger transmembrane glycoprotein receptor, is found in a variety of cell types, including tumour cells [30], adipocytes [31], macrophages [32], and certain epithelial cells. It is also involved in tumour metabolism, promoting metastasis and modulating FA absorption [33].

CD36, also known as fatty acid translocase (FAT), FA transport protein family (FATPs), solute carrier protein family 27 (SLC27), and plasma membrane fatty acid-binding proteins (FABPpm) are discovered to be highly expressed in tumours and are implicated in the FA *de novo* synthesis [34]. Exogenous FAs are activated to fatty acyl-CoA before being shuttled into the mitochondrion for FAO by the enzyme carnitine palmitoyltransferase I (CPT1) in the outer mitochondrial membrane. On the matrix side of the inner membrane, Carnitine Palmitoyltransferase II (CPT2) transforms fatty acylcarnitine to acyl-CoA. The final phase involves converting acyl-CoA to acetyl-CoA and its breakdown product via the TCA cycle, which is combined with oxidative phosphorylation to generate ATP. Aside from bioenergetic production, FAO-generated acetyl-CoA can be transferred to the cytoplasm via the TCA cycle and used to regulate cytosolic NADPH [27].

Despite the plasticity of metabolic circuits, macrophages preferred to have a distinct metabolic phenotype to meet their functional requirements. In general, macrophages favour certain metabolic pathways for energy production, primarily glycolysis and the pentose phosphate pathway (PPP). Other metabolic pathways such as glycolysis, PPP and the TCA cycle, can be used as precursors for *de novo* FA synthesis in addition to providing energy [35]. In macrophages, FAs and TAGs are generated through a sequence of enzyme processes known as *de novo* lipogenesis. Acetyl-CoA catalyses the formation of FAs, which are then converted to TAGs or phospholipids and incorporated into the plasma membrane or oxidised via the oxidation pathway [29].

Lipid biosynthesis is necessary for anti-tumoral M1 macrophages to alter the cellular membrane and produce pro-inflammatory mediators [36]. The metabolic activities of proinflammatory M1 macrophages have been thoroughly studied, and they rely not only on a high glycolytic metabolism to generate rapid ATP, but also on fuelling the TCA cycle to produce acetyl-CoA. (Fig. 1). On the other hand, ATP-citrate lyase converts glucose-derived citrate into acetyl-CoA. (ACLY). Infantino et al. found that ACLY levels increased rapidly in LPS-activated macrophages and that ACLY activity was suppressed, resulting in decreased amounts of NO, ROS, and prostaglandin E2 inflammatory mediators [37].

Besides ACLY, sterol regulatory element binding proteins (SREBPs) regulate lipogenesis, which is required for the production of FAs and cholesterol [38]. In LPS-induced macrophages, Srebp1-a, an isoform of SREBP, is widely expressed, and its activated genes encode for inflammasome components. A study discovered that mice lacking the Srebp1-a gene have a poor innate immune response [39]. FASN, like cancer cells, is a major enzyme in FA production in M1 macrophages, and it is primarily regulated by SREBPs. Carroll et al. demonstrated that the FASN intermediate metabolite, acetyl-CoA, contributes to the cellular process of lipopolysaccharides (LPS) even when they are not involved in palmitate production [40].

Anti-inflammatory M2 macrophages have an intact TCA cycle and metabolic changes that result in enhanced FAO and mitochondrial OXPHOS, both of which are driven by FA absorption [36]. FAs are taken up by M2 macrophages through lipolysis of circulating lipoproteins and FAs that are internalised via CD36 and can transcriptionally activate the nuclear receptors peroxisome proliferator-activated receptor gamma (PPAR) and peroxisome proliferator-activated receptor gamma coactivator-1 beta (PGC-1β) [41].

**Fig. 2:**
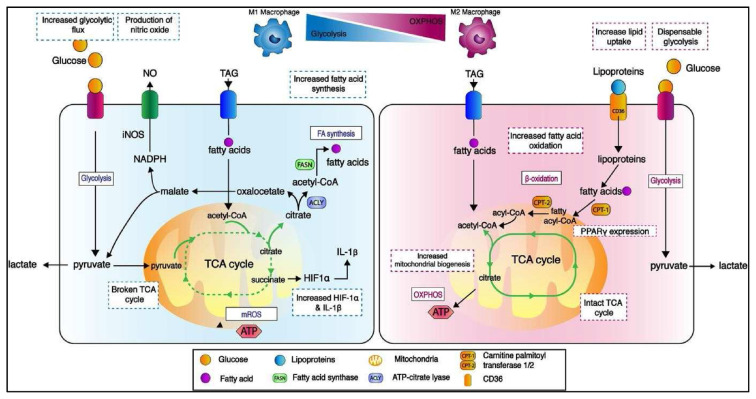
Metabolic control of M1 and M2 macrophage mitochondrial components is implicated in immunological activities. Changes in metabolic pathways such as the TCA cycle, oxidative phosphorylation (OXPHOS), and fatty acid oxidation (FAO) might result in completely different immune functioning profiles. Due to glycolytic flux is higher in M1 macrophages, the TCA cycle experiences two breakdowns resulting in elevated citrate levels. Citrate drives FA synthesis, which results in the formation of nitric oxide (NO) and prostaglandins. The second break occurs, resulting in higher succinate levels which stabilises hypoxia-inducible factor 1-alpha (HIF-1) and, as a result, increases interleukin-1 beta (IL-1) production and inflammation. On the other hand, M2 macrophages use a metabolic pathway that is fueled by FAs and OXPHOS, which are critical for M2 macrophage anti-inflammatory action. Due to glycolysis is hampered in M2 macrophages due to reduced HIF-1 activity, M2 macrophages redirect FAs toward re-esterification and FA-oxidation. Lipolysis of circulating lipoproteins and FAs, which are internalised via CD36, boosted lipid absorption in M2 macrophages. The stimulation of OXPHOS in M2 macrophages is demonstrated mechanistically by the expression of peroxisome proliferator-activated receptor gamma (PPAR). NADPH stands for nicotinamide adenine dinucleotide phosphate, acetyl-CoA stands for acetyl-Coenzyme A, fatty acyl-CoA stands for fatty acyl-Coenzyme A, acyl-CoA stands for acyl-Coenzyme A, FASN stands for fatty acid synthase. Triglyceride, triglyceride, triglyceride, triglyceride CPT-1: carnitine palmitoyltransferase-1; CPT-2: carnitine palmitoyltransferase-2; mROS: mitochondrial reactive oxygen species; CPT-3: carnitine palmitoyltransferase-3; CPT-4: carnitine palmitoyltransferase-4; CPT-5: carnitine palmitoyltransferase.

FAs are taken up by M2 macrophages via lipolysis of circulating lipoproteins and FAs that are internalised via CD36 and can transcriptionally activate the nuclear receptors peroxisome proliferator-activated receptor gamma (PPAR-β) and peroxisome proliferator-activated receptor gamma coactivator-1 beta (PGC-1 β) [42]. The PPAR activation in M2 macrophages mechanistically regulates the activation of the oxidative programme in these cells (Fig. 2). As indicated by the transcription of M2 macrophage characteristic genes in response to OA and IL4 stimulation, PPARs function as FA sensors and transcriptional activators of the FAO enzyme [43]. PPAR has recently been linked to the regulation of integrin β3-mediated M2 polarisation, with integrin β3 decreasing M2 polarisation by inhibiting PPAR overexpression [44]. Due to FA generation is required to fuel FAO and FAO plays a critical role in M2 activation, Huang et al. discovered that TAG substrate uptake via CD36 and subsequent lipolysis by lysosomal acid lipase (LAL) is more expressed in M2 macrophages than in M1 macrophages and is required for complete macrophage M2 activation upon IL-4 induction [45]. These data suggested that exogenous TAG absorption and lipolysis may contribute to FAO production during M2 activation.

## 3. Molecular mechanisms of fatty acid metabolism in cancer metastasis

Cancer metastasis is a complex process that has received a great deal of attention. Cancer cells spread from initial lesions to distal organs via extravasation from blood vessels, efficient infiltration in the bloodstream, colonisation of a new metastatic niche, tumour angiogenesis formation, and escape from a lethal immune-cell conflict. The invasion-metastatic cascade occurs as a result of cancer cells infiltrating or partnering with stroma, escaping immunosurveillance by immunoediting effector cell functions, evading and modifying the TME, and evolving drug resistance.

Cancer cells become aggressive and migrate from the epithelial origin borders into the surrounding stroma when metastatic progression begins [46]. Therefore, the epithelialmesenchymal transition (EMT) process has emerged as an important mode of metastatic potential. EMT can also be defined as a series of well-coordinated changes in which cells become immobile and strongly connected to the adjacent extracellular matrix (ECM), resulting in changes in FA metabolism [47,48]. Tumour cells with a mesenchymal phenotype and epithelial-mesenchymal phenotypic plasticity are more effective in circulating and priming for secondary site colonisation and metastatic formation [49].

FASN enzyme is involved in the endogenous synthesis of PA and its overexpression has been linked to poor prognosis, recurrence, and aggressiveness in a variety of cancers [50]. According to Li et al., FASN enhanced EMT in MCF-7-MEK, human breast cancer cell lines via regulating liver fatty acidbinding protein (L-FABP), VEGF, and VEGFR-2 [51]. Overexpression of FASN protein most likely resulted in an excess of long chain FA production, which was then transported to the cell membrane by L-FABP. This also causes EMT to occur because the ligand VEGF influences the position of the membrane protein receptor, VEGFR2, via lipid rafts [52]. A recent comprehensive proteome study using isobaric tags for relative and absolute quantitation (iTRAQ)-based spectrometry discovered the FASN-interacting protein network, which is thought to be important in the regulation of hepatocarcinoma invasion and metastasis [53]. The bioinformatics technique discovered four proteins (FSCN1, SIPA1, SPTBN1 or CD59) that interact with FASN and govern hepatocarcinoma invasion and metastasis, potentially by regulating EMT and MMPs.

Cancer cells that successfully EMT become circulating cancer cells (CCCs) before spreading and entering the bloodstream. CCCs’ ability to survive and colonise new niches is determined by their interactions with microenvironmental stimuli. The hybrid glycolysis/OXPHOS metabolic phenotype gives numerous benefits to cancer cell metabolic adaptability. Nonetheless, it was shown that CCCs may promote distant metastasis by adapting to the lymph node (LN) microenvironment through the use of many FAs. Yes-associated protein (YAP) is specifically activated in LN-metastatic tumours, resulting in the overexpression of genes involved in the FAO signalling pathway for metabolic shift to FAO [17].

While some ECM remodelling occurs often in the premetastatic niche, further changes inside the metastatic niche are required to promote cancer cell spread. The FA metabolism is an important process influenced by hypoxia [54]. Normal cells predominantly rely on external FA uptake, whereas cancer cells aggressively reactive *de novo* FA synthesis, regardless of circulating FA levels [55]. Cells developing under normoxia create unsaturated fatty acids (UFAs) from saturated fatty acids (SFAs) such as PA via oxygen-dependent desaturation. Another FA-related metabolic enzyme, stearoyl-CoA desaturase, regulates this process (SCD1). Therefore, hypoxic cancer cells have a deficient desaturation stage and rely heavily on UFAs produced from the environment to maintain cellular processes such as membrane formation and signalling [16,56].

SCD1 is a key lipogenic enzyme in the *de novo* synthesis of FA. It regulates tumorigenic events by converting saturated fatty acids (SFAs) such as palmitic acid (C:16) and stearic acid (C:18) into monounsaturated counterparts such as palmitoleic acid (16:1n-7) and oleic acid (C18:1n9) (Fig. 1) [57]. Several evidences suggested that SCD1 overexpression and MUFA anomalies were involved in the progression of a range of human malignancies, including prostate, colorectal, and lung cancers [58–60]. SCD1 activation is responsible for modifying intracellular SFA/MUFA ratios and is linked to the FA metabolism.

In human mesenchymal stromal cells (hMSC), SCD1 expression generated by liver X receptors (LXRs) resulted in a significant decrease in PA-induced cell mortality, caspase 3/7 activation, endoplasmic reticulum (ER) stress, and inflammation [61]. An epidemiological cohort study previously indicated that the FA desaturation index measured in blood lipids was associated to lower SCD1 activity and a lower risk of breast cancer [62]. These epidemiological data revealed why suppressing SCD1 expression inhibits breast cancer cell proliferation and invasion while also having a strong inhibitory effect on tumour development and growth. According to the data, both human serum and glucocorticoid- inducible kinase 1 (SGK1) have a similar role in the FA absorption and survival of NCI-H460 hypoxic human cancer cells. On the other hand, the addition of OA dramatically increased the survival of serum-deprived hypoxic NCI-H460 cells, whereas PA was severely hazardous to the cells [54].

PA has been shown to increase metastatic potential in a CD36-dependent manner. According to Pascual et al., PA increases the size and frequency of CD36-dependent lymph node metastasis in oral squamous cell carcinomas (OSCCs) and increases the metastatic potential of CD36+ metastasis without affecting primary tumour growth [30]. The discovery is consistent with the findings of Pan et al., who discovered that CD36 is involved in the clearance of lipids from the extracellular environment [63]. They found that phosphorylating AKT in gastric cancer cells causes glycogen synthase kinase 3 (GSK-3)/-catenin degradation and promotes CD36-regulated gastric cancer metastasis. It was also discovered that adding PA to oesophageal squamous carcinoma (ESCC) improves cell survival and works as an energy source, showing that CD36 is a vital fuel for cancer growth. When compared to essential amino acid treatment, Yoshida et al. found that PA therapy significantly increased cell viability in TE15 cells expressing CD36 [64]. Surprisingly, CD36-blocking antibodies generate a significant buildup of unmetabolized endogenously formed lipids, resulting in impaired metastatic lipotoxicity and cell death [30]. Their findings suggest that CD36+ metastasis starting cells rely on dietary lipids to drive metastasis in a CD36-dependent manner.

According to Wen et al., the activation of sterol regulatory element binding transcription factor 1 c (SREBP-1c) controls the expression of FASN and SCD1. SREBP-1c inhibition restored migratory and invasion abnormalities in AGS and SGC-7901 gastric cancer cells [65,66]. These enzymatic alterations reduced PA synthesis, which inhibited AGS and SGC-7901 cell motility and invasion in a concentration- dependent manner. OA enhances cervical cancer migration and growth via activating CD36-dependent activation of Src kinase and downstream of the ERK1/2 pathway [67]. Otherwise, the OA triggered the AMP-activated protein kinase (AMPK) pathway in highly metastatic cancer cells such as gastric carcinoma, HGC-27, and breast carcinoma, MDA-MB-231, which showed opposing responses to low metastatic cancer cells [68]. AMPK is an important mediator in regulating cellular energy homeostasis and helps in the maintenance of high ATP levels. When AMPK is activated, ATP levels are maintained while FA -oxidation is increased, implying that these high metastatic carcinoma cells may consume OA to support malignancy [69].

Given the prevalence of FAs synthesis pathways in cancer, the integration of podosomes and invadopodia coordinates the invasive and migratory capabilities of normal and tumour cells, respectively. As previously discussed, the FA synthesis pathway may create metabolic cues to facilitate cancer cell invasion and metastasis via membrane-mediated mechanisms such as podosomes and invadopodia. Exogenous palmitate (C16:0) and oleate (C18:1) can restore cell invadopodia by inhibiting acetyl-CoA carboxylase 1 (ACC1) activity [70]. The suppression of ACC1 causes membrane lipid composition to be disrupted. This is consistent with the involvement of FA metabolism and invadopodia, which have been related to tumour cell spreading potential via membrane–cytoskeleton interactions.

The imbalanced ratio of saturated to unsaturated phospholipids caused by accumulation of free cholesterol in the ER and exogenous addition of SFA have previously been shown to induce ER stress and initiate the unfolded protein response (UPR) [71,72]. Increased SFA accumulation inhibits SCD1 and induces cell death in glioblastoma cancer stem cells via an overactive ER stress response [73]. According to Huang et al., the end product of FASN activity, PA, significantly reversed the effect of α-mangostin ER stress and autophagy induction [74].

The composition and fluidity of the cellular membrane highlight the structural function and biological qualities of living cells, which include cell growth and division, receptor signalling, enzymatic activity, and cell–environment interaction [75]. Previous studies have shown that membrane fluidity is directly related to metastasis and the efficiency of newly discovered antimetastasis drugs is dependent on their capacity to reduce membrane fluidity. Membrane fluidity is known to be affected by the ratio of different FA chain lengths in the membrane bilayer and cell–cell contact. Lin et al. discovered that PA inhibits the formation of hepatocellular carcinoma (HCC) via inhibiting glucose absorption and energy metabolism [76]. Their proliferation data revealed that PA administration reduced LM3 cell membrane fluidity and ATP production, specifically reducing malignant cell proliferation and impairing cell invasiveness. Notably, the administration of PA to nude mice carrying LM3 cell carcinoma xenografts inhibited tumour growth and the creation of metastatic nodules.

Meanwhile, an *in vivo* study found that antimetastasis medication treatment reduced metastatic nodules, which may be reversed by restoring fluidity by OA injection. Membrane fluidizer OA treatment results in loose membrane packing and increased membrane fluidity [77]. However, antimetastasis drugs may reduce membrane fluidity in a variety of cell types. These studies demonstrated that a membrane’s lipid composition plays a significant role in regulating membrane fluidity, maintaining cellular activities, cell migration, and treatment resistance in cancer [78]. Intriguingly, cancer cells with high levels of lipid saturation and low membrane fluidity are found to be resistant to chemotherapeutics due to decreased drug uptake, highlighting the relationship between membrane fluidity and membrane permeability and implying a possible relationship between FA synthesis and drug resistance.

## 4. Fatty acid metabolism and metastasis aided by TAMs

The mechanism of TAMs in conjunction with their FA metabolism changes is also important in the successful metastasis of cancer cells. Macrophages within the TME contribute to a growth-suppressive state, although these cells may be reprogrammed by the tumour to exhibit pro-tumorigenic behaviours later on. On the other hand, TAMs promote phenotypic plasticity in the main tumour site, including invasion, vascularization, intravasation, extravasation, premetastatic niches, and CCC survival.

Metastatic tumour cells reconstructed their basal membrane by abandoning cell–cell junctions in order to cling to the surrounding tissue structure loosely. A great number of studies have found that bidirectional metabolic communication between tumour cells and TAMs plays an important role in the regulation of the EMT process. Biologically, numerous soluble molecules implicated in the EMT process including as IL-1, IL-8, tumour necrosis factor (TNF), and transforming growth factor-β (TGF-β), are secreted by macrophages via activation of acyl-CoA synthetase, which catalyses the thioesterification of FAs [79]. As a result of chemokines and bidirectional interactions, the tumour niche creates an immunosuppressive environment by attracting other cells. The inhibitory programmed death-1 receptor (PD-1) mediate immunological checkpoint in T cells will be activated by their ligands, PD-L1 and PDL2, which are produced by TAMs. An *in vivo* study by Lau et al. showed that the PD-1 ligand pathway modulates anti-tumour immunity with TAM-derived PD-L1 contributing to immunosuppressive responses [80].

CCC dissemination is particularly facilitated by close interaction with macrophages. Recent studies suggest that infiltrating CD163+ TAMs (M2 phenotype) trigger EMT to increase colorectal cancer migration, invasion, and metastasis through modulating the JAK2/STAT3/miR-506-3p/FoxQ1 axis, which leads to the production of CCL2 for macrophage recruitment [81]. TAM-assisted metastasis is facilitated by TAM proteolytic enzymes such as MMPs, cathepsins, and serine proteases, which are critical for ECM breakdown and cell-ECM interactions [82–84].

According to Robblee et al., IRE1 is required for PA therapy to activate the NLRP3 inflammasome [85]. ER stress and NLRP3 inflammasome activation are important in the TLRmediated signalling cascade, allowing macrophages to secrete proinflammatory cytokines, including IL-1 (Fig. 2) [86]. This will stimulate FAO in inflammatory macrophages, which will later create an anti-inflammatory impact. It is hypothesised that increased FAO favours M2 macrophages over M1 phenotypes. Several investigations have found that some M2 macrophages rely on FAO for proinflammatory and pro-migratory effects via FAO-associated ROS generation and an NLRP3-dependent mechanism, which could stimulate IL-1 transcription via hypoxia-inducible factor 1-alpha (HIF-1) upregulation [87].

In contrast to PA, most studies have reported that OA directly modulates PA-induced metabolism, ER stress, and inflammatory signalling suppression. OA reduces inflammation by decreasing PA-secreted IL-1 and inhibiting the NLRP3 inflammasome [88]. Similarly, oleatepalmitate TAGs have been shown to reduce SFA-induced ER stress and different proinflammatory palmitate metabolites [89]. OA inhibits intracellular crystallisation, NLRP3 inflammasome activation, and SFAs-triggered IL-1, such as PA and stearic acid [90].

Notably, the participation of macrophages in cancer is complicated by their heterogeneity and phenotype. Lipid droplets contribute to macrophage polarisation or phenotype, respectively, and oleate alone is sufficient to induce TAMs at phenotypic and functional levels, as well as induced substantial lipid droplet production in human CD14+ monocytes [91]. Wu et al. revealed that, in addition to cytokine signalling, increasing lipid metabolism, particularly oleate, is sufficient to convert CD206+MHCII^low^ myeloid cells into M2-like protumoral macrophages. The expression of M2 markers such as Retnal, Arg1, Chile3l3, and CD206 on both mRNA and protein levels was seen in these oleate-polarized CD206+MHCII^low^ myeloid cells.

Aside from these findings, TAMs discovered in a mouse mammary cancer were reported to have significant levels of epidermal fatty acid binding protein (E-FABP), an intracellular lipid chaperone that increases antitumor action [92]. Thus, it is inferred that E-FABP is mostly expressed in the M1-like macrophage population, and that these E-FABP expressing macrophages have a higher IFN-β response via lipid droplet accumulation in response to unsaturated FAs and tumours. This mechanism promotes the recruitment of tumoricidal effector cells, which boosts anti-cancer activity in the TME [93]. These findings suggested that macrophages could use tumour-derived lipids to boost antitumour action in response to unsaturated FAs like OA by upregulating E-FABP.

Nitric oxide (NO) is an important modulator of macrophage function, and its expression is linked to macrophage cytotoxicity against transformed cells [94]. Abnormal cells within the TME including macrophages, neutrophils, endothelial cells, fibroblasts, and in some cases, tumours, may produce NO. Despite its well-established anti-tumorigenic qualities, NO has been shown to have a dual function, whereby NO expression stimulates tumour development in some circumstances in a concentration-dependent manner. The potential of NO to modulate metastatic cell functions such as cell death, adhesion, motility, secretion, invasion, and angiogenesis explains this apparent discrepancy [95].

For instance, macrophage killing mechanisms regulated by high NO production to cause apoptosis of established tumour cells and cell cycle arrest. On the other hand, low NO concentrations protect cells from death [96]. Therefore, a high level of NO has been linked to the activation of an apoptotic response in tumour formation, and macrophages may use it as a weapon in their arsenal to penetrate and limit the growth of cancer cells. Monmai et al. found that PA was the most abundant SFA isolated from the tunic of *Halocynthia aurantium*, that it increased the levels of pro-inflammatory cytokines and immunological signalling for immune enhancement, and that it inhibited NO generation for anti-inflammation in macrophages [97]. According to Monmai et al. and Kim et al., OA strongly repressed a dose-dependent generation of TNF-, suppressed iNOS expression, and consequently decreased NO production in cultured cells. Furthermore, these hampered COX-2 induction, which was inconsistent with a full reduction of NO in culture medium. This suggests that OA inhibits NF-B binding activity in PC12 cells by preventing IB-breakdown [98]. Rudnicki et al. provided the first evidence that nitro-fatty acids (NO2-FAs), which include nitro-oleic acid-induced endothelial cell migration and sprout formation, stimulated angiogenesis *in vivo* in a NO-dependent way [99].

## 5. Conclusions

The modification of critical fatty acids in cancer metabolism has a significant impact on cancer invasion and metastasis. FA metabolism is a network of pathways with plasticity and bidirectional metabolic communication that has been fine-tuned to satisfy the metabolic needs of cancer cells. Metabolic intermediates by cancer cells can be exploited as an energy source to fuel their rapid progression, prevent membrane formation, and modify phenotypic to prime metastatic potential, finally leading to aggressive malignant progression. In macrophages, the differences in FAs metabolism between M1 and M2 macrophages have merged as an exciting topic, and metabolic alterations of each type of macrophage correspond closely to their cell function and phenotype, as well as their implications in pathophysiological lipid-related disease like cancer. Another topic to explore is identifying metabolic intermediates implicated in FA metabolism in both cancer cells and macrophages, which leads to altered cell expansion and tumour formation. We would be able to identify and expand the repertory of actionable metabolic regimes, as well as more precisely target metabolic communications, as we continue to investigate metabolic communications, particularly in the metastatic niche.
